# Towards Cultural Adequacy of Experience-Based Design: A Qualitative Evaluation of Community-Integrated Intermediary Care to Enhance the Family-Based Long-Term Care for Thai Older Adults

**DOI:** 10.3390/healthcare11152217

**Published:** 2023-08-07

**Authors:** Thin Nyein Nyein Aung, Thaworn Lorga, Saiyud Moolphate, Yuka Koyanagi, Chaisiri Angkurawaranon, Siripen Supakankunti, Motoyuki Yuasa, Myo Nyein Aung

**Affiliations:** 1 Department of Family Medicine, Faculty of Medicine, Chiang Mai University, Chiang Mai 50200, Thailand; drthinnyeinaung@gmail.com (T.N.N.A.); chaisiri.a@cmu.ac.th (C.A.); 2 Global Health and Chronic Conditions Research Group, Chiang Mai University, Chiang Mai 50200, Thailand; 3 Faculty of Nursing, Chiang Mai Rajabhat University, Mae Hong Son Campus, Mae Hong Son 58000, Thailand; thaworn.lorga@gmail.com; 4 Department of Public Health, Faculty of Science and Technology, Chiang Mai Rajabhat University, Chiang Mai 50300, Thailand; saiyudmoolphate@gmail.com; 5 Department of Judo Therapy, Faculty of Medical and Health Sciences, Tokyo Ariake University of Medical and Health Sciences, Tokyo 135-0063, Japan; koyanagiy@tau.ac.jp; 6 Centre of Excellence for Health Economics, Faculty of Economics, Chulalongkorn University, Bangkok 10330, Thailand; siripen.s@chula.ac.th; 7 Department of Global Health Research, Graduate School of Medicine, Juntendo University, Tokyo 113-8421, Japan; moyuasa@juntendo.ac.jp; 8 Faculty of International Liberal Arts, Juntendo University, Tokyo 113-8421, Japan; 9 Advanced Research Institute for Health Sciences, Juntendo University, Tokyo 113-8421, Japan

**Keywords:** ageing, caregiving, Community-Integrated Intermediary Care (CIIC), older adult, Thailand

## Abstract

In this qualitative study, we provided an in-depth understanding of how Community-Integrated Intermediary Care (CIIC), a new service model for family-based long-term care (LTC), was perceived by its users. The CIIC, established in Chiang Mai, Northern Thailand, consisted of three main interventions: (1) A temporary respite care center; (2) A family-centered care capacity building; (3) Functional training delivered as community group exercise and home exercise to improve healthy ageing for independent older adults. Ten pairs of dependent Thai older adults, their primary family caregivers, and ten village health volunteers were recruited using the purposive sampling method. Data were collected via semistructured in-depth interviews. A thematic descriptive qualitative analysis was used for data analysis. The findings revealed that CIIC helped reduce the burden of family caregivers by providing respite, relief, and care coordination. The experiences of the CIIC users indicated possibilities for service redesign, development, and delivery strategies to better meet the LTC needs of older adults and family caregivers. Following the local stakeholders’ commitment and local community health volunteers’ network, a well-integrated formal and informal care CIIC model can be implied as an effective and sustainable ageing care service model in Thailand and other Asian countries in the future.

## 1. Introduction

Population ageing is increasing worldwide [1,2] and Thailand is expected to step into the super-aged society as the percentage of Thailand’s total population aged over 60 years old is estimated to reach 28% by 2031 [3]. The sustainability of the traditional family-based long-term care model, currently practiced in Thailand and other Asian countries, has been challenged by the rapidly ageing population, increasing skipped generations where grandparents are left at home with their grandchildren to take care of each other reciprocally, and increasing direct and indirect cost from informal caregiving by the family members [4,5,6,7]. To address these challenges in sustainability through human resources and technical needs, a randomized controlled trial, Community Integrated Intermediary Care (TCTR20190412004; Thailand (CIIC project)) was implemented in Chiang Mai where 18.2% of the total population comprised older adults [8].

The CIIC center was established in February 2020 on a campus of the Mae Hia Municipality, Chiang Mai, Northern Thailand. This new service model, serving as an intermediary care center, was situated and integrated within the local community. The intermediary care concept would link families and communities to formal local older adult care services and funding within each municipality. The CIIC center, an intervention component of the research trial, was a result of the coordinated efforts between Japanese and Thai academics, local government, and health care providers [9,10]. The Japanese team offered technical and financial support, while the Thai team allowed the use of physical spaces and established the needed mechanisms for the operation of the CIIC center. The efforts were aimed at responding to the challenges imposed by the lack of innovative and alternative long-term care (LTC) services amidst an increasing number of Thai older persons and families with complex LTC needs. The CIIC project was set out to provide the following services: screening and assessment of family caregiver burden and LTC capacity, care capacity building for family caregivers, care prevention exercise program for independent older persons, and respite care service for families having difficulties with caregiving for dependent older adults with functional limitations, measured by using Barthel’s activities of daily living (ADL). The primary outcome was to reduce family caregivers’ burden. The secondary outcomes included improvements in ADL, depression, and quality of life of older adult participants.

### 1.1. Physical Spaces of the CIIC

The center was set up in a building adjacent to the main building of the Mae Hia Municipality. The CIIC room measured 6 × 4 m^2^ and was accessible via a main entrance door and an exit door at the rear. It was furnished with four beds, a dining table, and two recliners. Optimal room temperature was maintained by two air conditioners and two fans. Mobile curtains were provided to maintain privacy when needed. A toilet specifically designed for people with mobility impairments was installed.

### 1.2. The COVID-19 Pandemic and the Implementation of the CIIC Project

Despite a very solid implementation plan, the CIIC project could not be fully implemented due to the COVID-19 pandemic in Thailand. With the emergency declaration on 26 March 2020 [11], and COVID-19 pandemic containment measures, we stopped the activity from March until the end of June 2020. For safety concerns of the people and all parties involved in the implementation, the community-based care prevention group exercise activities were replaced by a home exercise program. A home exercise DVD, poster, and dairy calendar were provided by the CIIC project. Home visits and technical training to enhance the care capacity of family caregivers in need were also interrupted by social distancing measures during the COVID-19 pandemic. However, the research team continued to monitor the burdened family caregivers and provided tailor-made technical guidance via an online platform. The implementation was reassessed from time to time due to the COVID-19 situation. Unfortunately, as the pandemic remained serious and unpredictable, the CIIC center’s ability to accept dependent older adults with burdened family caregivers was put on hold indefinitely for safety reasons and to comply with the government measures to curb infection rates. To plan for reimplementation and to ensure the best fit of the CIIC services postpandemic, we conducted a qualitative study to gauge unique experiences of older adults, families, and service providers. Taking the experience-based design (EBD) approach [12,13,14], we aimed to identify the implications for improving or revising the originally planned CIIC services. The EBD approach is a method of capturing experiences and designing better service encounters leading to satisfaction and wellbeing for those using and delivering health and social services. Despite CIIC not being implemented fully, we aimed to explore the understanding of older adults, families, and stakeholders’ views on CIIC as it is necessary to estimate the extent of their acceptability of CIIC and to identify other potential benefits of the CIIC center apart from those highlighted in the proposal.

## 2. Materials and Methods

### 2.1. Ethics, Study Design, and Participants

This study was conducted following the Declaration of Helsinki [15]. The World Health Organization Ethical Review Committee (WHO/ERC ID; ERC.0003064, dated 7 March 2019) and Ethical Review Committee for Research in Human Subjects, Boromarajonani College of Nursing Nakhon Lampang, Praboromarajchanok under the Institute for Health Workforce Development, Ministry of Public Health, Thailand (approval number E 2562/005, dated 4 March 2019) approved the ethics of the study. It has been registered at the Thailand Clinical Trial Registry, trial registration number TCTR20190412004. We conducted face-to-face in-depth interviews, using semistructured questionnaires with 10 pairs of Thai older adults and their primary family caregivers and 10 village health volunteers (VHVs) via door-to-door home visits. Older adults have been defined as participants aged 60 years completed and above. The primary family caregiver is defined as the family member who spends the most time providing unpaid care to the dependent care recipients and who is perceived by themselves and others as the principal person responsible for caring for older adults. Village health volunteers, the heart of primary healthcare works, have been playing a key role as community health workers in the primary healthcare system in Thailand for many decades [16]. Based on the Thai cultural ideals of volunteerism and helping each other, VHVs are key community personnel in the delivery of care for older adults with chronic diseases [17]. By using the purposive sampling method, potential participants were approached and asked by a community volunteer if they were interested in participating in the interview. Those who expressed their interest were notified of the nature of their participation in the study and they were advised that their participation in the study would not have any impact on their current or future provision of healthcare. All the participants that were approached agreed to participate in the project and consented to their participation. Each participant was interviewed once at their home by a trained qualitative researcher. To improve quality assurance and to avoid investigator bias, qualitative inquiry and analysis were led by an external expert. The families and older adults were shown a set of pictures of the CIIC center, which was easily accessible and located within their local community. Afterward, we asked them to reflect on specific issues such as how they liked the ideas of CIIC, what they expected from the CIIC center, their contributions to the services, and their preferences for the services and the physical structure of the room. The caregiver interview was guided by a series of open-ended requests and questions: (1) Please tell me of your experience (in the past and at the present) with caregiving of the older person; (2) What are the negative and positive sides of your caregiving? (optional); (3) Please tell me of your experience with CIIC and its utilization; (4) What should we do to improve your caregiving experience? (5) What should we do to make CIIC work for you? The older adult participant was simply asked “In what regards do you think the CIIC has helped you and your family?” The VHVs were asked about their roles in CIIC, the roles of CIIC in long-term care, and the improvements needed. Following their responses to these guided questions, additional in-depth questions were used to elicit their responses in detail.

### 2.2. Data Analysis

In-depth interview transcripts were audio-recorded and transcribed into English. Participants were deidentified and assigned study-specific codes to exclude their names and any other identifying details in any study data electronic file to secure the confidentiality of participation. Data analysis was carried out via manual qualitative analysis using thematic charts [18,19,20,21]. Trustworthiness of qualitative analysis was ensured according to the four criteria identified by Lincoln and Guba [22]: credibility (e.g., frequent debriefing sessions among researchers); transferability (e.g., comparison with previous literature); and dependability and confirmability (e.g., using replicable methods, detailed description of the study protocol). Thematic descriptive qualitative analysis of the data took the following steps. For each interview transcript, two researchers read through the whole transcript to capture the holistic picture of the participant’s experience. These two analysts then identified the smallest units of data together and assigned them a meaningful code (called open coding). The open codings with similar meanings were then grouped into subthemes. Lastly, subthemes were grouped into a theme. The two analysts discussed subthemes and themes to maximize the best fit between the themes and subthemes and their respective coding of the participant’s quotes. The analysts then checked whether the themes and subthemes reflected the participants’ experiences. These themes and subthemes were modified via subsequent analyses. As a result, there were four themes and eleven subthemes to identify the additional features of CIIC to meet the needs of Thai older adults, their families, and other stakeholders for the future implementation of CIIC.

## 3. Results

The study participants included bedridden older adults (Barthel’s ADL score 0–4), with the most common underlying condition of stroke, followed by cancer, dementia, and hip fracture. Primary family caregivers are mostly constituted of spouses and children with a mean age of 55.27 ± 13.7 years. The VHVs were mostly middle-aged well-to-do persons with their own businesses, such as grocery shop owners, booksellers, food kiosk owners, florists, etc. Four themes were identified from interviews with family caregivers, Thai older adults, and volunteers. The themes are as follows: (1) Becoming a good caregiver; (2) Stretching to the limit; (3) Taking care of one’s health; (4) The roles of CIIC in addressing gaps in care and strengthening care systems. A summary of the emergent themes, subthemes, and participants’ quotes is described in Table 1.

### 3.1. Theme 1: Becoming a Good Caregiver

Caregivers of older adults in need of LTC at home have gone through a recognizable pattern of caregiving experiences. It is important to note that, in most cases, only one family member was selected as the principal caregiver. The sole principal caregiver had to shoulder the caregiving responsibility and tasks throughout their developmental career as a caregiver. Family caregivers began with being assigned a responsibility to care for dependent older adults regardless of their readiness or preparation to take on this duty of care. They may or may not be adequately prepared by a hospital or healthcare facility. As a result of the healthcare providers’ lack of knowledge of the whole picture of the situations and contexts in which care was taking place, most of these existing services could not meet the information and instrumental needs of the care recipients and their families. Therefore, informational, educational, and training services tended to be one-size-fits-all services. It was very common for family caregivers to seek additional information and knowledge to provide proper care to their dependent older adults. This often resulted in caregivers receiving incomplete, untailored (too general), and conflicting information which made it difficult for them to use the knowledge in managing care. Apart from the information, caregivers also had to acquire the skills required to work with older adults. These skills included skills related to medical procedures, moving, or transferring the care recipients, waste management, nutrition and hydration management, hygiene care, self-care assistance, and communication. The process of mastering caregiving is not static. It is rather a dynamic process as it is dependent upon the changing needs of older adults as their illnesses change. The vast knowledge and skills the family caregivers have received either through health service providers or self-inquiries are put into play. The acquired knowledge and skills were sometimes applied through a trial-and-error process. This process may take weeks or months before the caregivers can figure out what works best for the patient, for them, and their family. Knowing the care recipient was one of the critical aspects for the quality of care for older adults with complex LTC needs. As the caregivers became more involved and engaged with their caregiving, they observed the care recipients and learned about their behaviors, problems, and needs. They also learned what works and what does not. Knowing the care recipients not only helped increase the quality of care but also reduced caregivers’ frustration because of trial and error. It also improved the caregivers’ confidence and morale as they have achieved the desired outcomes of care.

### 3.2. Theme 2: Stretching to the Limit

To meet the needs of older adults, family caregivers must stretch their limits. The situation was particularly stressful for families living with very limited incomes, irregular jobs and earnings, and a limited number of capable caregivers. This also applied to caregivers having ill health and chronic conditions which need to be managed. Everything was centered around the care recipient and their needs were prioritized over other needs of the caregivers. Caregivers were trying their best to put the needs of their care recipients ahead of theirs. Healthcare providers also focused solely on the needs of the dependent older adults without extending specific considerations to the needs of caregivers or families. Healthcare assessments were purely directed at the care recipients, leaving very limited room for discussions about the caregivers’ family situations and wellbeing. Having to stretch their limits can negatively impact the family’s functionality, wellbeing, and the quality of care provided to their care recipients. Negative impacts of caregiving on different aspects of their life were noted, one of which was loss of a job due to voluntary job resignation to become a caregiver. In addition, others experienced a loss of economic productivity as they had to juggle between jobs and caregiving tasks. They also experienced muscle pains and aches, emotional stresses, social isolation, and lack of sleep.

### 3.3. Theme 3: Taking Care of One’s Health: Healthy Lifestyles Applied and Better Health Realized by the Caregivers

Despite evidence of negative experiences of caregivers about caregiving, they also reported positive aspects of caregiving. Being a caregiver for a certain period made the caregiver and the family health conscious. The caregivers learned about healthy lifestyle choices, applied what they learned by themselves, and realized the benefits of adopting healthy lifestyles. Understanding healthy foods and diets was the most common benefit highlighted by the primary family caregivers.

### 3.4. Theme 4: Roles of CIIC in Addressing Gaps in Care and Strengthening Care Systems

Caregivers experienced fragmentation in existing health services, especially during the early stages of caregiving. The early stages of caregiving often involved decision-making on health services, settlements of jobs and caregiving demands, and caregiving arrangements. This stage featured high emotional stress among caregivers due to the abrupt changes and crises in the family. Caregivers found it difficult to sort out all the information in a way that eased their decisions. Having multiple healthcare providers also contributed to the fragmentation of care as there was no overall governing body responsible for the LTC of older adults. There were also no specific focal point service providers who could navigate the caregivers into the system or liaise the services across different care systems. A stark fragmentation was found between health and social service systems. Bridging the two systems needed to be fulfilled by the families themselves. This often-caused unnecessary stress on the caregivers and delayed access to the required services and help. In the absence of comprehensive social services, caused by a lack of an effective coordination system between health and social care, dedicated and devoted volunteers became critical in liaising between the two systems. A dedicated and devoted volunteer is valuable and instrumental to care management. Volunteers helped communicate with the local government about the unmet needs of the care recipient older adults and caregiving families, raised community awareness about the families in need of support, conducted regular visits to their houses and assessed the ongoing problems and needs, connected families to existing resources within and outside the community, assisted the families to the extent of job and income generation, and spoke for the families. Their dedicated efforts have seen older adults receive the needed services from the local government such as home modifications and financial assistance. The proposed CIIC and its services were very well appreciated and accepted by families, older adults, and volunteers. The CIIC center was viewed as an alternative to respite care, not a replacement for family care. Home-based care is still the best option for families and older adults as the care recipients and caregivers prefer to be cared for in their own homes. Being in their own home is associated with having control of what they do, conveniently accessing needed items, and being less stressed due to the familiar surroundings and people at home. CIIC respite service is the perfect option when the family cannot juggle between routines and caregiving tasks. Respite care has a short-term stay nature, not a long-term continuous stay, and it is free of charge. Voluntary donations to the center should be considered an alternative to fixed-cost copayment. Proactive and outreach home-based services by CIIC were beneficial to older adults and their families.

## 4. Discussion

**Figure 1:**
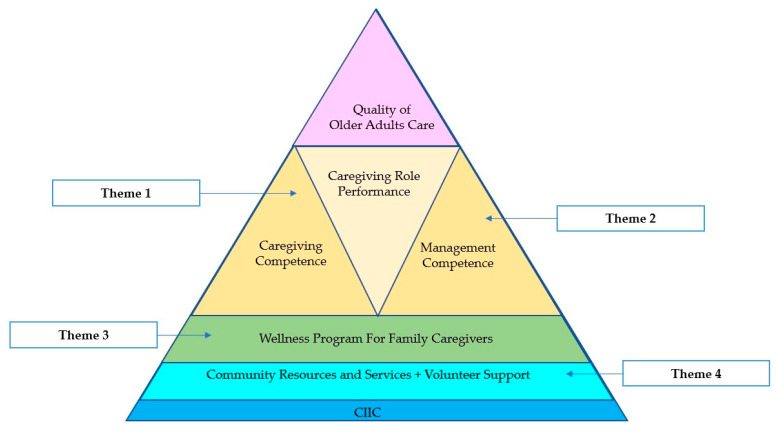
The Community Integrated Intermediary Care (CIIC) model, enhancing the quality of care for older adults.

In summary, this new service model suggests that CIIC bridges the gaps in existing health and social services and strengthens existing community resources such as volunteer caregiver support. To better address the experiences of service users and family care providers, services for family caregivers should include the following: (1) A wellness program for family caregivers; (2) Caregiving competence development; (3) Management competence development; (4) Ongoing support of caregiving role performance. The outcome of this model is quality of care (in terms of care for older adults and family caregiver outcomes such as family caregiver wellbeing) (Figure 1).

Emerging themes and subthemes resulting from the qualitative analysis can be implied for CIIC as follows.

### 4.1. Implications of Theme 1: “Becoming a Good Caregiver” for CIIC

In consideration of the four functions of the CIIC project ((1) Screening and assessment of family burden and LTC capacity; (2) Care capacity building for family caregivers; (3) Care prevention exercise program; (4) Respite care service for families having difficulties with caregiving), we suggest that CIIC considers both principal and supplemental or other types of caregivers in capacity building. These caregivers can help share the caregiving responsibility and help offset the principal caregivers’ burden and negative impacts. CIIC provides ongoing and continuous support to family caregivers and their care recipients. The support should respond to changing needs of caregiving which are developmental in nature and very dynamic across different stages of caregiving and illnesses [23,24]. Regular assessments of the needs of caregivers should also be an essential part of this ongoing support. Supports for caregivers may be guided by the developmental career of the caregiving framework.

### 4.2. Implications of Theme 2: “Stretching to the Limit” for CIIC

Services provided by CIIC take a family-centered approach and have dual focuses. They emphasize the needs of the care recipients and those of the caregivers. CIIC should assist health and social care providers to better understand and effectively provide family-centered services. This may be achieved through formal or informal training and workshops [24,25,26]. As caregiving-related stresses can be quite detrimental to the health and wellbeing of both dependent older adults and caregivers, measures to respond to their needs in a timely and adequate manner should be explored and considered by the service providers [25,27,28,29,30]. A range of innovative health and social services are suggested to be offered to caregivers. These include, and are not limited to, health promotion, illness prevention, income generation, financial assistance, counselling, social and religious activities, and respite care.

### 4.3. Implications of Theme 3: “Taking Care of One’s Health” for CIIC

CIIC should reap the opportunity to introduce health promotion services to caregivers and families. Family caregivers are aware of staying healthy not only for themselves but also for their care recipient who is really in need of his/her help all the time. These health promotion services include the promotion of exercise activities, the introduction of a balanced diet to improve nutrition, and avoidance of substance abuse such as smoking and alcohol.

### 4.4. Implications of Theme 4: “Roles of CIIC in Addressing Gaps in Care and Strengthening Care Systems” for CIIC

To address the fragmentation in formal health and social services, CIIC should be involved in care management as early as possible to prevent unnecessary caregiving-related stresses and delays in accessing healthcare services. Navigation and liaison services may be considered to help bridge the gaps between different service providers [31,32]. Amidst the current lack of a designated formal care coordinator, a dedicated volunteer can be assigned and trained to serve as a care coordinator [33,34]. These volunteer coordinators work with families, social care providers, health providers, and other community organizations to ensure that older adults and families can access the needed services on a timely basis [35]. The lessons from CIIC noted that dedicated and devoted volunteers are valuable and instrumental in care management. They are advised to be equipped with the mindset, knowledge, and skills to effectively perform the assigned roles. Moreover, volunteers can be recognized as an integral part of CIIC and the wider community care systems. Their contribution to the systems should be formally and informally valued and recognized on various important community platforms. Moreover, CIIC is considered acceptable as it is a well-integrated home-based and CIIC center-based service. Alternatives to the pay-for-service approach should be explored with consideration of the abilities of the families to pay and the existing financing mechanisms of health and social services. The finding of this study to reflect the usefulness and acceptability of CIIC via qualitative evaluation may be limited by the Thai culture of consideration for others and feeling of gratitude and reciprocity. However, the strength is that our findings may form the tip of the iceberg where sustainability and maintenance of implementation are required to see the full effectiveness to provide stronger evidence for an ageing world.

## 5. Conclusions

Our findings supported that the experience-based design of CIIC could help family caregivers of older adults reduce caregiver burden and become good caregivers by providing ongoing and continuous support for care capacity building via a family-centered approach. In addition, CIIC also provided a respite care center to host the dependent older adults when their family caregivers are burned out or not available for caregiving temporarily. In consideration of sustainability, traditional home-based and CIIC-based services should be well integrated. A welfare-free-of-charge approach to service financing may be considered, depending on the ability of families to pay for the services. By using effective community support and local stakeholders for the provision of infrastructure for ageing care and a strong community health volunteers’ network, the model of CIIC can be sustainable for healthy ageing communities in Thailand.

## Figures and Tables

**Table 1:** A summary of the emergent themes and participants’ quotes in qualitative descriptive analysis.

	Themes Summary	Participants’ Quotes
Theme 1	Becoming a good caregiver	
Subtheme 1	A responsibility to care: family caregivers began with being assigned a responsibility to care for dependent older adults regardless of their readiness or preparation to take on this duty of care.	“*He is my husband. It’s my responsibility to take care of him. It can’t be the others.*” (FCG ID 1)
		“*I am tired of caregiving, but I am happy as I must return for spending her life to take care of her children when we were young.*” (FCG ID 2)
		“*It’s a family responsibility. We can’t deny it or expect others to do it for us.”* (FCG ID 8)
Subtheme 2	Mastering oneself as a caregiver: The process of caregiving mastering is not static. Rather, it is quite dynamic and dependent upon the changing needs of older adults as their illnesses change.	“*Every day is a learning day for me. I learned about the disease that he got. I learned about his medications. I learned everything that I need to learn to look after him well. I practiced and learned many skills to help him with daily tasks. It’s a lot easier now [than in the past] to help him through the day.”* (FCG ID 1)
		*“You get better at it [caregiving task] the next time you do it. I felt it was challenging at the beginning, but I think I’m pretty good at it now.”* (FCG ID 4)
Subtheme 3	Applying the knowledge and skills in caregiving: the acquired knowledge and caregiving skills are sometimes applied through a trial-and-error process.	*“The doctors and nurses would give us a lot of advice and knowledge. But we need to make it fit with the care recipient. It’s not always like what the doctors said to us.”* (FCG ID 4)
		*“Our house is very small, and it is packed with our stuff. There are steps all over the place. Getting around in a wheelchair inside the house is almost impossible. We need to use tandem walking quite a lot to help her move around the house. We tied a rope around her waist to make tandem walking easier and safe [so she would not fall].”* (FCG ID 3)
Subtheme 4	Knowing the care recipients: it is one of the critical aspects for the quality of care for older adults with long-term complex care needs.	*“He was barely talking. So, we had to guess what made him look unhappy and restless. We had to try different things to help him. Many things didn’t work. But finally, we would find something that worked for him. You need to know the older people. I think each person is unique. You must be a good observer and attentive to care for them well.”* (FCG ID 1)
		*“When she frowns and moves her legs, I know there must be something wrong with her. I would check whether she wet or soil herself first as this is often the case when she behaves like this.”* (FCG ID 10)
Theme 2	Stretching to the limit	
Subtheme 1	Everything is centered around the care recipient: the needs of care recipients are prioritized over other needs.	*“It’s always about her. It [caring for her] is the priority. We must settle her care first before we do something else.”* (FCG ID 2)
		*“Everyone asks about their patient but not so much about us [caregivers]. They would ask whether the care recipient progresses well or has any problems. I understand that we have very limited time during each consultation [at the hospital] so we need to focus on the care recipient.”* (FCG ID 3)
Subtheme 2	Meeting caregiving burdens and demands at all costs	*“We would do everything to keep him well. Anything that we can afford even though it is hard. Physically, financially, and emotionally.”* (FCG ID 9)
		*“My dad has been bedridden for about three years, and I used all my time and efforts to take care of him. It was very difficult for me to make proper positioning, transfer, and mobility before I got specific guidance from the CIIC team. Thank you and because of the improvement in caregiving skills, I can spare some time to take care of myself every day.”* (FCG ID 4)
		*“I need to sacrifice a lot. I had to quit my job in the city to be her caregiver. Now I don’t have a monthly salary. I opened a food kiosk at home so I can be with her and have some regular income.”* (FCG ID 3)
Subtheme 3	Impacts of caregiving on caregivers and family functioning	*“I suddenly became jobless when both of my parents got bedridden as I am the only child to take care of them. I did not feel guilty about quitting my job, but I was exhausted from caregiving.”* (FCG ID 8)
		*“I don’t have time to myself. In fact, the whole family doesn’t have time for us. We have no life outside our home. No more gatherings with friends. It can be tough sometimes.”* (FCG ID 9)
		*“There are ups and downs all the time. This was especially at the beginning of her illness. A lot was happening then. There were times when I felt that it was too much. I am coping much better now but I can expect a time when I feel low.”* (FCG ID 2)
		*“We have to juggle the care tasks and other things. It’s a constant planning and decision making.”* (FCG ID 7)
Theme 3	Taking care of one’s health	
Subtheme 1	Healthy lifestyles applied and better health realized by caregivers	*“Taking care of others has taught me a lot about the importance of having good health. If you don’t want to go down that path [being bedridden], you must look after your health very well. I am careful about what I eat. I try to go for a walk whenever I have time.”* (FCG ID 5)
		*“I could get advice from the CIIC team not only for specific caregiving skills such as bed bathing, occupied bed making, etc. but also nutrition advice on how to prepare a balanced diet for my mother”.* (FCG ID 2)
		*“I have my blood pressure taken by the volunteer as often as I can. I weigh myself at least once a week, so I know what my body is like.”* (FCG ID 8)
		*“One of the good things about being a caregiver is that I have better health. My muscles are much firmer now, and I feel a lot healthier because of following the healthy lifestyle.”* (FCG ID 10)
Theme 4	Roles of CIIC in addressing gaps in care and strengthening care systems	
Subtheme 1	Fragmentation in formal health and social services: having multiple health providers also contributed to fragmentation in care as there was no overall governing body responsible for the long-term care of older people.	“*There were many hurdles that we had to go through to get things we wanted for the older adult. We [the family] had to navigate the [health and social] services by ourselves. The services were there but we needed to connect them. To do that, we had to communicate with different people again and again. You know, there are two separate systems that we must go through. It’s not a smooth process at all.”* (FCG ID 3)
		*“Information from different health providers can be inconsistent. It’s up to us to decide which one is right for us. No one knows the whole picture of the care recipient. They seem to know parts of it, so it’s hard to get everything done in one go.”* (FCG ID 4)
Subtheme 2	A dedicated and devoted volunteer is instrumental in care management	*“Without the volunteer, our family would not be able to stand on our own feet now. She is so supportive. She is always there for us. She helps talk to the local government and visits us often. She is the one who cares enough to ask us how we are.”* (FCG ID 1)
		*“She is the one who makes sure patients and families in need of support get help. She has a strong sense of community where everyone cares for everyone. She helps promote our foods in the community so that we can have a good sale and earn more money.”* (FCG ID 4)
		*“I am proud of myself to be a health care volunteer to assist burdened families with dependent older adults. I assumed that this is our duty to take care of each other in the local community.”* (Volunteer ID 4)
		*“I am a retired person and I have plenty of free time to take care of my neighbors. I am happy to contribute some good things to my village.”* (Volunteer ID 1)
		*“When I noticed that she locked her father in bed, I know it is not good care. I think it is our responsibility to help burdened families in our community.”* (Volunteer ID 6)
		*“I am happy as I can do CIIC exercise and encourage my friends to do so.”* (Volunteer ID 8)
Subtheme 3	Acceptability of the CIIC: The proposed CIIC and its services are very well appreciated and accepted by families, older adults, and volunteers. The CIIC center is viewed as an alternative to respite care, not as a replacement for family care. Home-based care is still the best option for families and older adults.	“*Thank you. I feel pity for my son for taking care of me all the time. I want him to take a break. I am pleased to hear that I can stay there for one week.”* (Older adult ID 4) *“It is located within our village. I think I can stay happily there for a short time but not for a long time.”* (Older adult ID 5) *“I don’t want to stay there. I have decided to stay at home until my last breath. Please come to help my daughter who is tired of caregiving.”* (Older adult ID 8) *“Is it expensive? I noticed she is using a lot of money for buying things for me including diapers and skin lotions. If it is free of charge care, I want to stay there for a while.”* (Older adult ID 9) *“When I heard about the CIIC project where we can send our dependent care recipients for a short-term stay, I felt a big relief, even though I could not have a chance to use their respite care service because of the COVID-19 situation.”* (FCG ID 8)
		*“I think free-of-charge care can be difficult to sustain long term. You better charge the rich family to use your CIIC center services.”* (FCG ID 3)
		*“I like the idea of CIIC center as it is located within our village and my mother may not feel being away from home.”* (FCG ID 7)
		*“I felt encouraged when I heard that I could send my mother to your care center temporarily. Thank you.”* (FCG ID 10)

Note: FCG = Primary Family Caregiver.

## Data Availability

The data presented in this study are available on request from the corresponding author.
